# The Effect of Curcumin on the Differentiation of Mesenchymal Stem Cells into Mesodermal Lineage

**DOI:** 10.3390/molecules24224029

**Published:** 2019-11-07

**Authors:** Armita Mahdavi Gorabi, Nasim Kiaie, Saeideh Hajighasemi, Tannaz Jamialahmadi, Muhammed Majeed, Amirhossein Sahebkar

**Affiliations:** 1 Research Center for Advanced Technologies in Cardiovascular Medicine, Tehran Heart Center, Tehran University of Medical Sciences, Tehran 1411713138, Iran; armitamahdavi61@gmail.com (A.M.G.); breeze.nasim@yahoo.com (N.K.); 2 Department of Medical Biotechnology, Faculty of Paramedicine, Qazvin University of Medical Sciences, Qazvin 15315-34199, Iran; saeideh.ghasemi67@yahoo.com; 3 Halal Research Center of IRI, FDA, Tehran, Iran; jamiat931@mums.ac.ir; 4 Department of Nutrition, Faculty of Medicine, Mashhad University of Medical Sciences, Mashhad, Iran; 5 Sabinsa Corporation, East Windsor, NJ 08520, USA; mail1@samilabs.com; 6 Biotechnology Research Center, Pharmaceutical Technology Institute, Mashhad University of Medical Sciences, Mashhad, Iran; 7 University of Western Australia, Perth 6009, Australia; 8 Neurogenic Inflammation Research Center, Mashhad University of Medical Sciences, Mashhad, Iran

**Keywords:** curcumin, stem cell differentiation, mesenchymal stem cells, mesodermal lineage

## Abstract

Curcumin has been placed at the forefront of the researcher’s attention due to its pleiotropic pharmacological effects and health benefits. A considerable volume of articles has pointed out curcumin’s effects on the fate of stem cell differentiation. In this review, a descriptive mechanism of how curcumin affects the outcome of the differentiation of mesenchymal stem cells (MSCs) into the mesodermal lineage—i.e., adipocyte, osteocyte, and chondrocyte differentiation—is compiled from the literature. The sections include the mechanism of inhibition or induction of MSCs differentiation to each lineage, their governing molecular mechanisms, and their signal transduction pathways. The effect of different curcumin doses and its structural modifications on the MSCs differentiation is also discussed.

## 1. Introduction

Curcumin, also known as diferuloylmethane or [1*E*,6*E*]-1,7-bis[4-hydroxy-3-methoxyphenyl]-1,6-heptadiene-3,5-dione, is a natural hydrophobic polyphenol derived from the rhizomes of the *Curcuma longa* plant. Alongside being used as a food-coloring agent, it possesses several pharmacologic effects such as antioxidant, anti-inflammatory, anti-microbial, antifungal, antiviral, anti-angiogenic, anti-atherosclerotic, and anti-cancer properties [1,2,3,4,5,6,7]. The pleiotropic pharmacological effects of curcumin have resulted in a wide range of formulations, which are available as nutritional supplements [8]. Despite its popularity as a pharmacological agent, its utility is limited due to its poor bioavailablity, absorption, and short half-life. To overcome these issues, different nanocarriers in the form of polymer conjugates, polymeric particles, lipid particles such as micelles, nanogels, magnetic nanoparticles made from liposomes, cyclodextrin, chitosan, and gold nanoparticles are being developed with variable success rates [9,10,11,12].

Curcumin has been shown to have beneficial effects on diseases such as obesity, osteoporosis, osteolysis, and osteosarcoma, in which stem cell differentiation plays an active role [13]. An excessive differentiation of mesenchymal stem cells (MSC) to adipocyte lineage results in an increased number of adipocytes (hyperplasia), and adipose tissue accumulation in obesity. During bone remodeling, osteoclasts resorb the mineralized matrix of the broken bone to prepare a favorable condition for osteoblast differentiation, while an excessive action of osteoclasts leads to bone loss and osteoporosis [14,15,16,17,18]. The beneficial effects of curcumin in obesity and osteoporosis suggest a plausible role of curcumin in the differentiation of MSCs into the mesodermal lineage, including osteoblasts, adipocytes, and chondrocytes.

MSCs are one of the major cell types used in regenerative medicine. Bone marrow or adipose tissues of individuals are major harvesting sites of MSCs. These multipotent stem cells give rise to a wide array of cells including bone, fat, and cartilage tissues in response to various regulators such as vitamin D3, bone morphogenic proteins, and osteogenic growth peptide to keep the balance of the body composition. Studies on the differentiation of MSC into these lineages is important for the specific application in bone and cartilage tissue engineering. Therefore, in this review, the effect of curcumin on the differentiation of MSCs into adipocyte, osteocyte, and chondrocyte, as well as the proposed mechanisms of these effects are discussed. A list of biomolecules mediating the process of mesodermal differentiation of MSCs following curcumin treatment are summarized in Table 1.

## 2. Importance of Pharmacokinetic of Curcumin for Its Pharmaceutical Effects

One major concern regarding the clinical use of curcumin is its low systemic bioavailability. The glucuronidation and sulfation of curcumin are two common processes that restrict its bioavailabilty after oral administration. After in vivo administration, curcumin undergoes first-pass and second-pass metabolism, and metabolites of curcumin, including curcumin glucuronide, curcumin sulfate, tetrahydrocurcumin, hexahydrocurcumin, and hexahydrocurcuminol are excreted from feces, urine, and bile [19]. Theses metabolites are also found in the cell suspensions during in vitro cell culture experiments with curcumin. In human studies, ingestion of 2 g of pure curcumin resulted in the presence of only 10 ng/mL curcumin in plasma [20]. The low bioavailability of curcumin necessitates using a high dose of oral curcumin to induce hemeoxygenase-1 (HO-1) expression, which is an important mediator of the antioxidant effects of curcumin and a regulator of the osteogenic differentiation of cells following curcumin exposure. Klickovic et al. showed that the consumption of even 12 g of curcumin C3 Complex^®^, a curcuminoids preparation with a patented ratio of curcumin I (curcumin; molecular formula: C21H20O6; molecular weight: 368.380 g/mol); curcumin II (demethoxycurcumin; molecular formula: C20H18O5; molecular weight: 338.354 g/mol); and curcumin III (bisdemethoxycurcumin; molecular formula: C19H16O4; molecular weight: 308.328 g/mol) was not associated with detectable curcumin in plasma (detection limit: 1 ng/mL) following oral administration and had no effect on HO-1 mRNA expression [21]. Oral administration of the same dose (12 g) of C3 Complex^®^ curcuminoids to healthy subjects in another study led to pharmacokinetic parameters such as AUC to be 26.57 ± 2.97 μg/mL × hr, Cmax to be 1.73 ± 0.19 μg/mL, tmax to be 3.29 ± 0.43 hr, and t1/2 to be 6.77 ± 0.83 hr [22].

## 3. Curcumin’s Effect on Adipogenic Differentiation

Curcumin has been reported to show beneficial effects in controlling obesity [23,24]. Weisberg et al. have reported reduced lipid content and body weight in curcumin-fed obese diabetic mice. In this study, a 95% standardized curcumin extract was orally given to the mice through meals of 4% fat by weight containing a 3% by weight of curcumin [25]. The anti-obesity effect of two doses of curcumin (2 µM and 10 µM) was reported to be mediated by its anti-inflammatory property and by inhibiting adipogenic differentiation [26]. The underlying mechanisms involved in the regulation of adipogenic differentiation by curcumin are discussed in detail in the subsequent sections.

### 3.1. Inhibition of Adipogenic Differentiation

**Figure 1:**
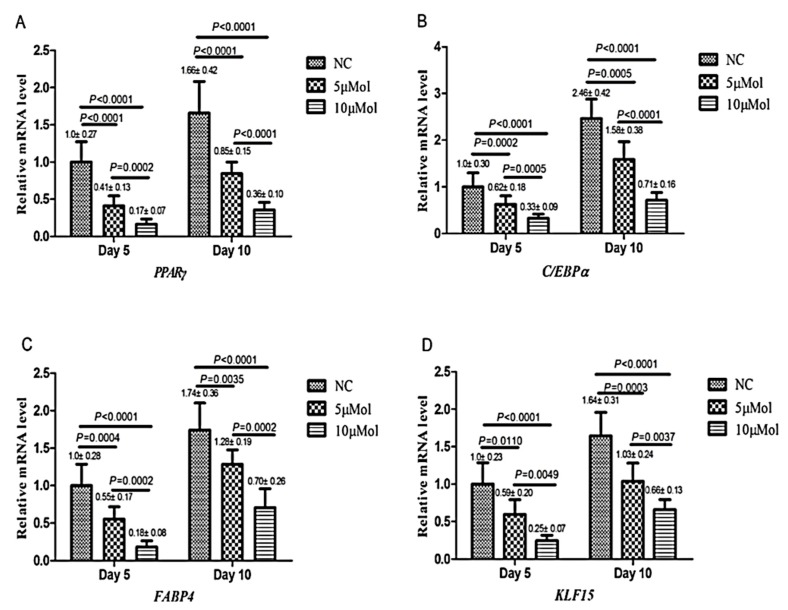
Curcumin’s inhibitory effect on the mRNA expression of adipocyte markers and KLF15. qRT-PCR analysis of the mRNA expression of PPARγ (**A**), C/EBPα (**B**), FABP4 (**C**) and KLF15 (**D**). All of the values are expressed as the mean ± S.D. (*n* = 3). All the experiments were independently repeated at least three times. Adopted with the permission from Wang et al. with license number of4595960186796 [28].

Curcumin was reported to inhibit the adipocyte differentiation of MSCs or mouse 3T3-L1 cells (a preadipocyte cell line) [27]. The adipogenic differentiation of cells is commonly characterized by the accumulation of lipid vesicles in the cytoplasm, as well as the expression of adipogenic marker genes such as peroxisome proliferator-activated receptor-γ (PPARγ), CCAAT/enhancer-binding protein-α(C/EBPα), fatty acid-binding protein (FABP)-4, and Kruppel-like factor 15 (KLF15). The role of KLF family members on adipogenic differentiation is diverse, in which KLF4 and KLF5 promote adipogenesis, whereas KLF2 and KLF3 suppress it [27]. Curcumin treatment was found to decrease the intracellular lipid droplet as observed by oil red O staining and the reduced expression of adipogenic markers via the mechanisms described below (Figure 1).

#### 3.1.1. AMPK Modulation

The first mechanism through which curcumin plays its role in adipogenic differentiation is by modulating adenosine monophosphate-activated protein kinase (AMPK). Curcumin is known as an AMPK activator similar to the synthetic AMPK activator, AICAR [29]. AMPK is a sensor of cellular energy status, and its activation exerts an inhibitory effect on adipogenic differentiation. According to studies, curcumin increases the phosphorylation of AMPK and acetyl-coenzyme A carboxylase (ACC), which suppresses the conversion of acetyl CoA to malonyl CoA. The activation of AMPK also inhibits glycerol-3-phosphate acyltransferase-1 (GPAT-1) expression and reduces fatty acid esterification. Carnitine palmitoyltransferase-1 (CPT-1) and GPAT-1 are the two enzymes involved in fat metabolism. Therefore, curcumin through AMPK activation directly affects lipid metabolism, and the phosphorylation of AMPK abrogates the expression of preadipocyte transcription factors including KLF15, PPARγ, C/EBPα, and FABP-4, as well as their proteins. PPARγ and C/EPBα expression synergistically stimulate adipogenesis, and FABP-4 regulates lipid storage and metabolism. Therefore, inhibiting the expression of these transcription factors prevents adipogenic differentiation [27,30].

#### 3.1.2. Wnt Signaling Pathway Activation

Activation of the Wnt/β-catenin signaling pathway by curcumin is the second mechanism for the inhibition of adipogenic differentiation. This pathway was suggested based on observations that curcumin increases the gene expression of Wnt receptors such as Wnt10b, frizzled (Fz2), and low-density lipoprotein receptor-related protein 5 (LRP5), as well as Wnt targets such as c-Myc and cyclin D1. Increased Wnt receptor expression activates the receptor-mediated signal transduction pathways, and drives β-catenin phosphorylation. Wnt/β-catenin signaling activation inhibits the phosphorylation of mitogen-activated protein kinases (MAPK), such as extra-cellular-regulated kinases (ERK), c-Jun N-terminal kinase (JNK), and p38. Since these MAP kinases are essential for the differentiation of 3T3-L1 cells to adipocyte, the administration of curcumin (10 or 25 µM) to 3T3-L1 cells via dissolving curcumin in a differentiation medium containing DMEM, 10% fetal bovine serum (FBS), 0.25 µM dexamethasone, 0.25 mM 3-isobutyl-1-methylxanthine (IBMX), and 1 µg/mL insulin, decreases the expression of adipogenic marker genes [31,32,33].

Curcumin also affects key Wnt signaling pathway effectors such as the Tcf7l2 gene. On one side, it ameliorates Tcf7l2 expression and on the other side, it inhibits miR-17-5p expression, and the result of both is the suppression of adipogenic differentiation and the mRNA expression of five adipogenic differentiation markers, including FABP-2, C/EBPα, C/EBPβ, cell death-inducing DFFA-like effector a (Cidea), and PPARγ [26].

#### 3.1.3. Fatty Acid Synthase Inhibition

The third mechanism of suppression of adipocyte differentiation of 3T3–L1 cells by curcumin is through inhibition of fatty acid synthase (FAS), a critical metabolic enzyme for lipogenesis, by targeting malonyl/acetyltransferase domain of FAS [34]. Curcumin reduces not only FAS protein level, but also its enzymatic activity, which leads to down-regulation of the PPARγ expression [34].

#### 3.1.4. Interaction with PPARγ Receptors

The fourth suggested mechanism is the direct interaction of curcumin with the ligand-binding domain (LBD) of the PPARγ receptor, which plays a crucial part in the adipogenic differentiation of cells. Ferguson et al. showed that an exposure of curcumin up to the 30 µM to the cells at 0 h, 24 h, 48 h, or 72 h from the onset of differentiation causes a preferential binding of curcumin to the LBD of the PPARγ receptor [35]. This observation was confirmed by measuring the binding affinity of different phenolic compounds to the LBD of PPARγ in 3T3-L1 cells through in silico molecular docking assay. The measured binding energy value for curcumin was reported to be −4.86 kcal/mol. Higher energy (closer to 0) values show less favorable ligand–receptor interaction while in the case of curcumin, the value is low and negative, thus showing the high binding affinity to the PPARγ receptor. Cys285, Arg288, and Leu333 residues constitute the binding site of PPARγ to curcumin [36].

#### 3.1.5. Inhibition of Mitotic Clonal Expansion

The fifth mechanism is attributed to the anti-proliferative effects of curcumin. Mitotic clonal expansion (MCE) is a vital step in the early stage of adipogenic differentiation, before the G1/S phase transition. Curcumin acts via inhibiting cyclin-dependent kinase (Cdk), which is a cell cycle regulator and proliferation-related transcription factor that is necessary for G1/S development and inhibits the MCE. The S-phase kinase-associated protein-2 (Skp2) regulates the degradation of p27, which is an inhibitor of Cdk. Curcumin at doses less than 30 µM blocks Skp2 protein accumulation and therefore inhibits p27 protein degradation. Curcumin not only increases the p27 accumulation but also improves the half-life of the protein. An increased p27 amount and stability suppresses cell division and therefore suppresses the early stage of adipogenic differentiation [35,37,38]. Cdk2 phosphorylates both C/EBPβ, which is essential for the transcriptional activation of PPARγ, and C/EBPα, during the early stage of adipocyte differentiation [39]. Hence, the inhibition of Cdk2, in turn, prevents the expression of these adipogenic transcription factors.

## 4. Curcumin’s Effects on Osteogenic Differentiation

Many studies show evidence that curcumin is beneficial for enhancing bone mineral density, improving bone microarchitecture, protecting against ovariectomy-induced bone loss, and preventing osteoporosis and arthritis [40,41,42,43,44,45]. These findings suggest that curcumin may induce bone remodeling through an inhibition or induction of osteocyte differentiation.

### 4.1. Induction of Osteogenic Differentiation

Curcumin dissolved in 9% ethanol and diluted in water has been reported to increase the differentiation of osteoblast from precursor cells [46]. Curcumin increased the expression of bone-associated gene markers such as bone morphogenetic protein 2 (BMP-2), runt-related transcription factor (Runx2), and osterix in MSCs in vitro [46]. Curcumin supplementation increased alkaline phosphatase (ALP) activity as an early indicator of osteogenic differentiation, the number of mineralized nodules, and osteocalcin (OCN) expression, which are markers of mature osteoblasts [42,47]. Furthermore, curcumin was found to not only increase osteogenic differentiation but also improve its transdifferentiation. The enrichment of differentiation medium (DMEM with 15% FBS) of mouse embryonic fibroblasts with 15 µM curcumin dissolved in DMSO and co-treatment with human LIM mineralization protein 3 (hLMP-3), a positive regulator of bone formation, resulted in improved hLMP-3 osteogenic potency and cell transdifferentiation processes [48]. According to Jain et al., the controlled release of curcumin from polymer scaffolds resulted in the osteogenic differentiation of MC3T3-E1 pre-osteoblasts seeded onto the platform. ALP expression and the deposition of calcium phosphate minerals increased with curcumin release, and quantitative real-time PCR analysis showed an increased expression of osteogenic-related genes such as ALP, Runx2, OCN, osteopontin (OPN), and BMP2 genes [49]. Interestingly, 10–15 µM curcumin dissolved in DMSO and added to the culture media increased the osteoblast differentiation of MSCs in the early stages of differentiation [50]. With attention to the role of curcumin in the induction of osteogenic differentiation, the underlying mechanisms are discussed in the following section.

#### 4.1.1. Akt/GSK3β Signaling Pathway Activation

**Figure 2:**
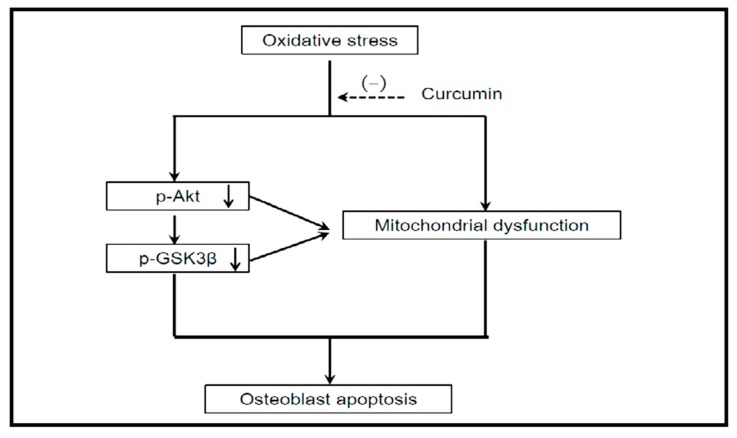
Working hypothesis: Curcumin administration ameliorated oxidative stress-induced apoptosis in osteoblasts through preserving mitochondrial function and activating Akt–GSK3β signaling. Adapted from Dai et al. [55].

One of the essential features of curcumin that affects osteoblast differentiation is its antioxidant and free radical scavenging potential [51]. Oxidative stress prevents the osteoblastic differentiation of MSCs [52,53]. However, the antioxidant properties of curcumin (0–25 µM) reduce oxidative stress and protect stem cells from oxidative injury, and thus consequently osteoblast apoptosis and osteoporosis [54]. Following oxidative damage, reactive oxygen species (ROS) generation and their accumulation in mitochondria facilitate the opening of the permeability transition pores and lead to mitochondrial dysfunction. Curcumin was found to decrease the mitochondrial oxidative status, and improve the mitochondrial membrane potential and functions. The antioxidant activity of curcumin reduces ROS generation and preserves the mitochondrial redox potential by increasing the levels of phosphorylated protein kinase B (Akt), which in turn phosphorylates glycogen synthase kinase-3β (GSK3β). Phosphorylated GSK3β is inactive, and thus the activation of caspase 3 is inhibited, protecting the osteoblasts from apoptosis (Figure 2) [55]. Curcumin has also been reported to possess a protective role against mitochondrial dysfunction in other tissues, including brain, liver, and kidney. Pathways such as PI3K/Akt seem to interfere with this protective mechanism [56,57,58,59,60].

#### 4.1.2. Maintaining Wnt/β-Catenin and Wnt/TCF Pathways

Wnt/β-catenin signaling plays a dominant role in osteogenesis. This pathway is a crucial modulator of osteogenic differentiation and is blocked by dickkopf-1 (Dkk-1), which is an extracellular inhibitor of Wnt signaling, resulting in a reduced osteogenic differentiation potential of cells [61]. The binding of ligands to the Wnt receptor activates a cytoplasmic cascade resulting in the suppression of GSK3β, which is a critical protein in the Wnt signaling pathway. GSK3β degrades β-catenin by forming a complex with this protein. In turn, the inhibition of GSK3β prevents the phosphorylation and proteasomal degradation of the co-transcription factor β-catenin, which can translocate to the nucleus and promote osteogenic differentiation through binding to T-cell factor (TCF) and initiating the Wnt/TCF pathway of osteogenic differentiation [62,63]. Curcumin ameliorates the expression of Wnt receptors such as Fz2 and coreceptors such as LRP 5/6 [31]. The antioxidant effect of curcumin also maintains the Wnt/β-catenin pathway via blocking the deteriorative effect of H_2_O_2_ (an important ROS signal) on β-catenin and its target protein cyclin D1, as well as C-myc, which are vital elements in Wnt pathway. Curcumin blocks H_2_O_2_ action on β-catenin via inducing ROS scavenger enzymes such as HO-1 [64]. Under oxidative stress, β-catenin acts as a cofactor in activating forkhead box O (FoxO) transcription. Hence, FoxO shows higher availability instead of TCF, and thereby the Wnt/TCF pathway is blocked. In turn, curcumin maintains the Wnt/TCF pathway by reducing oxidative stress, thus preventing the β-catenin from reacting with FoxO. Additionally, curcumin compensates for a decreased expression of C-myc and cyclin D1 and prevents the inhibition of the Wnt/β-catenin pathway [65].

#### 4.1.3. Keap1/Nrf2/HO-1 Signaling Pathway Activation

Up-regulating antioxidant gene heme-oxygenase-1 (HO-1) in MSCs by curcumin and activating kelch-like ECH-associated protein 1 (Keap1)/Nrf2 (nuclear factor erythroid 2-related factor 2)/HO-1 signaling is another way of reducing oxidative stress, improving resistance to oxidative stress, and improving osteoblast differentiation [42]. Curcumin, as a ligand, interacts directly with receptors on the Nrf2 protein via the hydrogen binding of Arg441 and Ile458 amino acids [66]. HO is an enzyme catalyzer for heme degradation. HO-1 expression and HO activity cause the osteogenic differentiation of MSCs [50]. Few studies report the increased expression of HO-1 in tumor cells, skin fibroblasts, hepatocytes, and cardiomyoblasts following curcumin exposure, and the activation of MAPK by curcumin is the underlying mechanism of increasing HO-1 expression [54,67,68,69].

Li et al. used curcumin to stop the overproduction of ROS in MSCs and promote osteogenesis. Curcumin was loaded into polylactic glycolic acid (PLGA) microspheres. Then, MSCs and curcumin-releasing microspheres were incorporated into a composite scaffold made from collagen and hydroxyapatite. The prolonged release of curcumin from the scaffold for 30 days resulted in decreased H_2_O_2_ production in diabetic serum. In addition, improved mitochondrial status was observed with curcumin treatment due to decreased nicotinamide adenine dinucleotide phosphate oxidase 4 (NOX4), and increased manganese-dependent superoxide dismutase (MnSOD) gene expression. Curcumin also activated the Keap1/Nrf2/HO-1 pathway by down-regulating Keap1 and up-regulating the total expression of Nrf2 and HO-1 genes. The activation of the Keap1/Nrf2/HO-1 pathway by curcumin in MSCs resulted in reduced ROS, improved osteogenic differentiation, and an increased expression of osteogenic markers including OCN, Runx2, and OPN [70].

#### 4.1.4. ER Stress Pathway Activation

**Figure 3:**
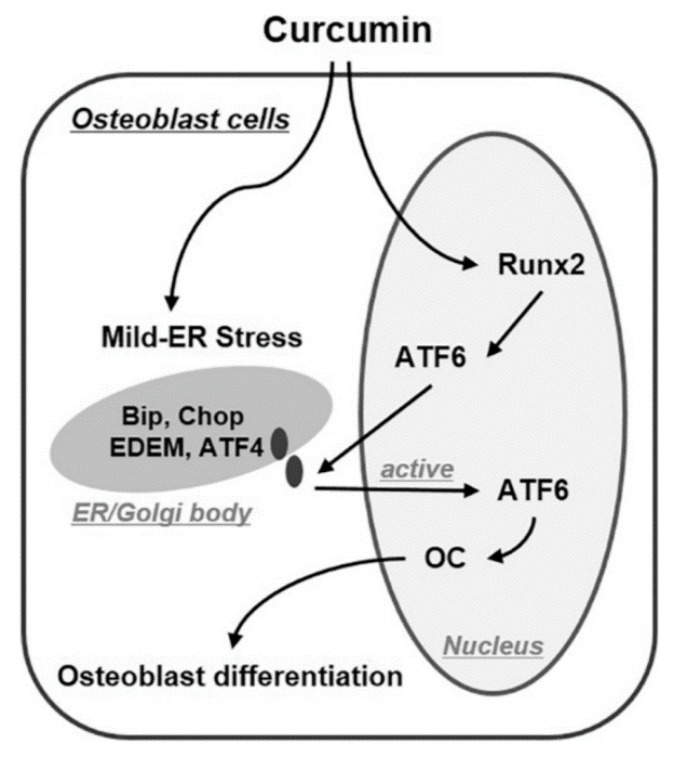
Proposed mechanistic model for osteoblast differentiation by curcumin treatment. Curcumin induced osteoblast differentiation through the regulation of ER stress levels in C3H10T1/2 cells (mesenchymal stem cells). Curcumin increased ATF6 expression and activation via Smad-mediated Runx2 expression, similar to BMP2 treatment. Furthermore, curcumin-activated ATF6 induced osteoblast differentiation by increasing osteocalcin (OC) expression. Adopted with the permission from Son et al. with the license number 4590160351301 [47].

The effect of curcumin on the osteoblast differentiation of MSCs is similar to that of BMP2, which is a cytokine in osteoblast differentiation. It activates the Smad 1/5/8 signaling pathway to regulate Runx2 expression and consequently osteogenic markers expression. For the stimulation of osteogenic differentiation, Smad-mediated Runx2 expression activates mild endoplasmic reticulum (ER) stress pathways involving primary unfolded protein response (UPR) inducers, such as protein kinase R (PKR)-like endoplasmic reticulum kinase (PERK), inositol-requiring enzyme 1 (IRE-1), and activating transcription factor 6 (ATF6). Curcumin, similar to the pathway mentioned for BMP-2, increases the expression of ER stress marker genes, such as immunoglobulin binding protein (BiP), CCAAT/enhancer binding protein (C/EBP) homologous protein (CHOP), ATF4, and ER degradation-enhancing-α-mannidose-like protein (EDEM). Along with this, the increased expression of ATF6 resulted in the increased OCN expression and osteogenic differentiation (Figure 3) [47].

### 4.2. Inhibition of Osteoblast Differentiation

There also exist few studies that claim the inhibitory effect of curcumin on osteoblast differentiation, and no study to date has investigated such effect in MSCs. Although, in the following section, the mechanism of inhibiting the osteoblast differentiation of fibroblasts and vascular smooth muscle cells (VSMCs) due to curcumin is presented.

#### JNK/Bax Signaling Suppression

Curcumin has been reported to decrease the osteoblastic differentiation of VSMCs by inhibiting apoptosis and calcification. The culturing of VSMCs in a media of elevated calcium and phosphate acts as a stimulator of the JNK/Bax signaling pathway. JNK, a member of the MAPK family, activates Bax, a cell apoptosis inducer, and thereby causes the apoptosis of VSMCs. In turn, the apoptotic bodies increase VSMC calcification and osteogenic differentiation. Additionally, elevated calcium and phosphate increase the expression of osteogenic markers such as Runx2, BMP2, and Osterix, as well as ALP activity. However, treatment with curcumin inhibits the JNK/Bax signal activation and decreased VSMCs calcification is observed as well [71].

### 4.3. Inhibition of Osteoclast Differentiation

Based on previous studies, it is clear that curcumin only exerts an inhibitory effect on the differentiation of MSCs to osteoclasts. The mechanisms involved in the inhibitory effect of osteoclast differentiation are discussed in detail in the following section.

#### 4.3.1. RANKL-Induced Signaling Pathway Inhibition

**Figure 4:**
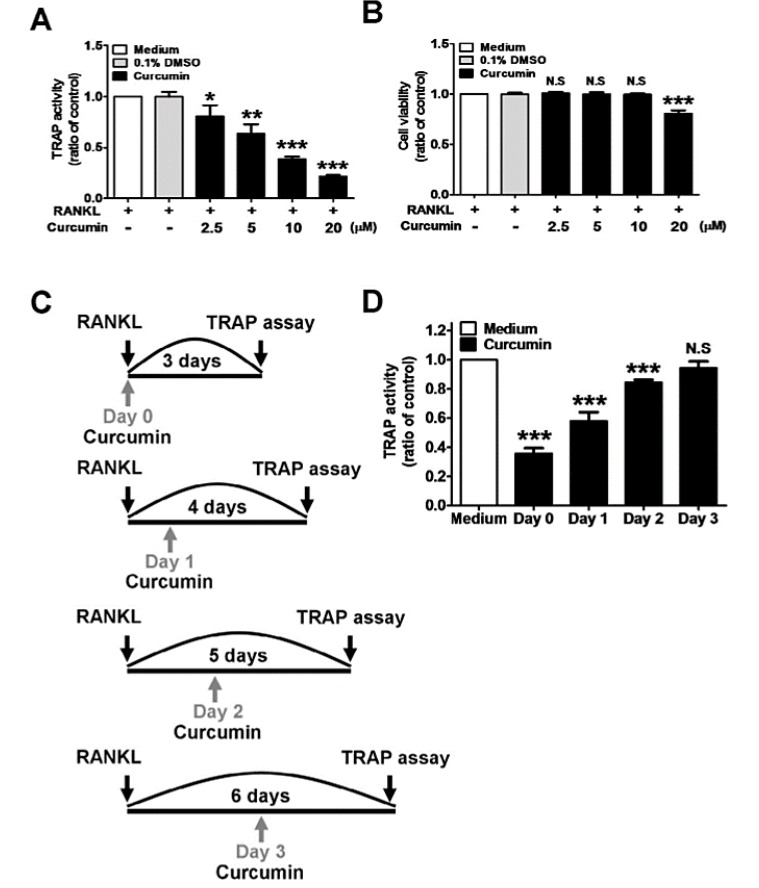
Curcumin inhibits the RANKL-induced osteoclastogenesis of RAW264.7 cells at an early stage. RAW264.7 cells (1 × 105 per well in a 24-well plate) were treated with an indicated concentration of curcumin in the presence and absence of 100 ng/mL RANKL. (**A**) Cells were lysed and reacted with buffer containing tartrate and p-nitrophenylphosphate (pNPP) solution. TRAP activity was analyzed using a spectrophotometer. (**B**) RAW264.7 cells were treated with curcumin in the presence and absence of RANKL. Cytotoxicity was determined using MTT assay. (**C**,**D**) RAW264.7 cells were treated with RANKL, and curcumin (10 M) was added at different time points (Days 0–3 after RANKL stimulation). TRAP activity was detected after adding curcumin following three days of culture. Data represent the mean ± SD of at least five independent experiments. *p 0.05, ** *p* 0.01, *** *p* 0.001 (Student’s t-test), different from values after treatment with RANKL alone. Adopted with permission from Mau et al. with license number 4595960699160 [72].

Nuclear factor κB ligand (RANKL) is an essential factor in the control of osteoclast function, and an increase in its expression depends on the oxidative stress. Due to curcumin’s antioxidant properties, the reduced amount of ROS consequently decreases the RANKL gene expression [65]. According to previous studies, low doses of curcumin (5–20 µM) showed a reduction in the H_2_O_2_-induced cell death of MSCs, recovered ALP activity, and up-regulated osteogenesis markers such as OPN and collagen 1 (COL1A1), whereas the expression of the RANKL gene was completely blocked [65]. Similarly, in bone marrow-derived monocytes (BMMs) and the mouse macrophage cell line (RAW 264.7 cells), treatment with RANKL and curcumin improved the tartrate-resistant acid phosphatase (TRAP) activity (Figure 4) and inhibited the RANKL-induced pathways. Treatment with curcumin down-regulated the gene expression of osteoclast differentiation markers such as c-Fos, nuclear factor of activated T cells 1 (NFATc1), TRAP, and osteoclast-associated immunoglobulin-like receptor (OSCAR). It also reduced the expression of genes such as dendritic cell-specific transmembrane protein (DC-STAMP), cathepsin K, and matrix metalloproteinase-9 (MMP-9), which is specific to osteoclast differentiation. In addition, it decreased the number of TRAP-positive multinucleated cells with three or more nuclei and the formation of actin rings [72,73].

In a study reported by Heo et al., curcumin loaded into gold nanoparticles inhibited the osteoclast differentiation of bone marrow-derived macrophages by reducing the activation of RANKL-induced signaling pathways. In this study, the formation of a RANKL-induced actin ring, a morphological feature of osteoclasts, was inhibited [74]. Liposome-loaded curcumin also inhibited RANKL-induced cathepsin K and TRAP’s gene expression and decreased the percentage of multinucleation and the TRAP activity in RAW264.7 macrophages [75].

#### 4.3.2. NF-κB Signaling Pathway Inhibition

The ROS-induced IκBα signaling pathway is another osteoclastogenesis route that is reported to be suppressed by curcumin due to its antioxidant properties. RANKL activates nuclear factor-kappa B (NF-κB) and its downstream NFATc1 signaling by various processes such as the phosphorylation, degradation, and kinase activity of IκBα. The suppression of ROS production by curcumin is reported to reduce IκBα degradation, thereby reducing the RANKL expression and inhibiting the activation of NFATc1, as well as the NF-κB signal pathway [73,76]. Curcumin exerts its inhibitory effect on NF-κB even before IκBα phosphorylation. Incubating ML-1a cells with curcumin (40–60 μM) inhibited tumor necrosis factor (TNF) and TNF-dependent NF-κB activation [77]. Curcumin also blocks NF-κB activation in cultured adipocytes [78]. An intraperitoneal injection of curcumin (300 mg/kg, dissolved in 1.0 mL corn oil) to the rats suffering from middle cerebral artery occlusion reduced NF-κB expression [79]. The inhibitory effect of some curcumin analogues, such as synthetic monoketone compound 3,5-Bis(2-fluorobenzylidene)-4-piperidone, is higher than curcumin so that one hour of exposure of a 1–100-μM compound dissolved in DMSO to the mouse RAW264.7 macrophages inhibited the NF-κB signaling pathway and TNF [80].

#### 4.3.3. RANKL/RANK Signaling Pathway Inhibition

With attention to curcumin’s anti-inflammatory effects, the suppression of osteoclastogenesis has been reported due to decreased proinflammatory mediators, such as cyclooxygenase 2 (COX-2) and MMP-3, and the blockage of interleukin (IL)-1β-induced inflammatory responses [75]. As a result of reduced inflammation, an increase in the osteoprotegerin (OPG) to RANKL ratio was observed in 7F2 osteoblastic cells treated with curcumin. The increased ratio indicates the inhibition of the RANKL/RANK signaling pathway [81].

#### 4.3.4. Wnt/β-Catenin Signaling Pathway Activation

Curcumin inhibits osteoclast differentiation through activating Wnt/β-catenin signaling. This pathway leads to the inhibition of β-catenin degradation. It is noteworthy that β-catenin regulates the expression of OPG as an inhibitor of osteoclast differentiation [82]. In addition, Wnt signaling is responsible for the repression of adipogenesis by the mechanism similar to osteogenic differentiation.

## 5. Curcumin Affects Chondrogenic Differentiation

The effect of curcumin on MSC cell differentiation to chondrocytes is less than the two other lineages, osteoblasts, and adipocytes. In chondrogenic differentiation, curcumin might act as an inhibitor or stimulator in function.

### 5.1. Induction of Chondrogenic Differentiation

Curcumin per se has no chondrogenic potential. However, it affects the chondrogenic differentiation of MSCs indirectly by suppressing inflammation [83]. Within the body, a superficial zone of articular cartilage is rich in MSC-like progenitor cells that are not able to differentiate into chondrocyte due to the high concentration of proinflammatory cytokines such as IL-1β at the site of cartilage injury and inflammatory reactions. Such inflammatory cytokines activate NF-κB, which leads to MSC apoptosis. The exposure of cells to 5 μM curcumin inhibits IL-1β-induced NF-κB activation and increases the level of collagen type II, cartilage-specific proteoglycans, and β1-integrin, which are characteristics of chondrogenic differentiation. The effect of curcumin on IL-1β is time and concentration-dependent. Curcumin also suppresses caspase-3 and prostaglandins such as COX-2, which are responsible for cell apoptosis and extracellular matrix (ECM) degradation [83].

### 5.2. Inhibition of Chondrogenic Differentiation

Reduced type-II collagen expression, inhibited cartilage nodule formation, and sulfated proteoglycan accumulation are all manifestations of the chondrogenic differentiation of MSCs, which are reported to be decreased after 48 h of exposure of 20 μM curcumin to cells at day 3 of differentiation [31]. In this case, curcumin inhibits chondrogenesis by either stimulating apoptosis or suppressing actin reorganization, which are two vital processes for chondrogenic differentiation by suppressing the phosphorylation of Akt, leading to Akt inactivation. Akt inactivation induces the actions of pro-apoptotic proteins such as Bax and stimulates cytochrome C release from the mitochondria, thus activating apoptosis. The other action of Akt inactivation is mediated by the down-regulation of integrin β1 and focal adhesion kinase (FAK) phosphorylation, thus stopping the integrin-mediated signal transduction, and resulting in actin reorganization [84].

## 6. Effect of Curcumin Dose on Mesodermal Differentiation of Cells

In different studies, various doses of curcumin are exposed to the cells or fed to the animals. In differentiation studies, curcumin mainly is dissolved in DMSO and then added to the cell culture or differentiation medium. For example, a 24-h incubation of cells with curcumin was done at different concentrations (5–20 mM) [30,38]. In animal studies, curcumin mainly is delivered through dietary supplementation. As an example, animals were fed with a high-fat diet (22%) supplemented with 500 mg of curcumin/kg for 12 weeks to study the adipogenic potential of curcumin [30]. A low dose (10 mg/kg) and high dose (50 mg/kg) of curcumin in the form of dietary spice was also administered to rats by a stomach tube daily for eight weeks with a limit of 20 g/day [44]. Doses of curcumin as high as 100 mg/kg was also delivered to rats orally for 30 days [46].

The stem cell differentiation and the inhibition/support of differentiation of MSCs by curcumin is reported to be dose-dependent. A dose of 10–15 µM curcumin is reported to suppress the adipocyte differentiation of MSCs, while the same treatment favors the osteogenic differentiation of MSCs [50]. Additionally, an optimization of curcumin dosage is necessary for planning a specific lineage differentiation of cells. For example, for the osteogenic differentiation of MSCs through the mechanism of reducing ROS generation, the curcumin dose should be optimized so that the levels of ROS necessary for osteogenic differentiation can be maintained [53].

In most studies of differentiation, the curcumin dose is around 10–50 µM, and the doses higher than 100 µM are reported to cause cytotoxicity [34]. Normally, doses higher than 20 µM create no significant difference in the induction of osteogenic or the suppression of chondrogenic differentiation [55,84]. Similarly, in the case of adipogenic differentiation, curcumin at 10 µM compared to 5 µM had a higher inhibitory effect on the expression of adipogenic proteins, suggesting its effect on the suppression of adipogenic differentiation to be dose-dependent [26,27,85]. In addition, doses of curcumin higher than 25 µM are reported to completely block adipocyte differentiation [30,31,34,35,85].

In case of osteogenic differentiation, curcumin with varying concentrations up to 20 µM shows an inhibition of osteoclast differentiation in a dose-dependent manner, while the optimal dose for the inhibition of osteoclast differentiation was found to be 10 µM [48,72,73,74]. This optimum curcumin dosage is also reported to up-regulate HO-1 expression, induce ER stress markers, and finally, induce osteoblast differentiation [47,50]. In an experimental periodontitis study, curcumin was evaluated for the inhibition of osteoclast differentiation, reduction of alveolar bone loss, and periodontal destruction. In this in vivo study, two doses of curcumin—50 mg/kg/day and 30 mg/kg/day—exerted protective effects on alveolar bone loss [72]. In the case of chondrogenic differentiation, 0–5 µM curcumin was reported to suppress IL-1β-induced apoptosis in a concentration-dependent manner [83].

## 7. Effect of Modified Forms of Curcumin on Mesodermal Differentiation

**Figure 5:**
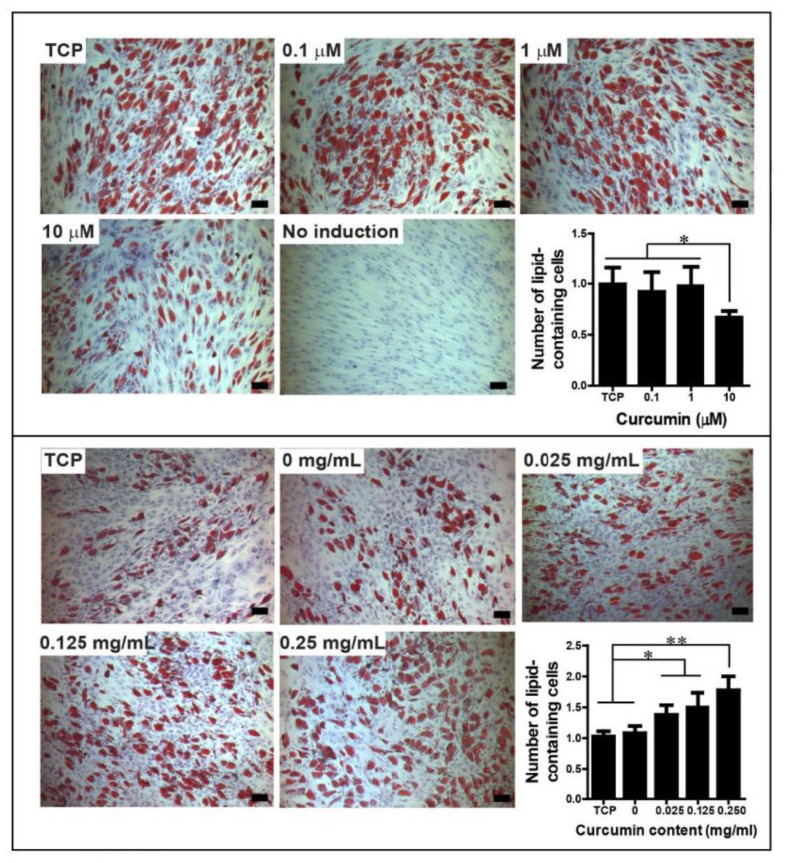
Human bone marrow-derived mesenchymal stem cells (hBMSC) adipogenic differentiation by oil red O staining. Upper panel, hBMSCs cultured in free curcumin-containing medium, day 14; Lower panel, hBMSCs cultured on curcumin-loaded silk hydrogel films, day 14. The number of lipid-containing cells was normalized to TCP control, N = 4–7. *indicates significant difference (*p* 0.05); ** indicates very significant difference (*p* 0.005). Scale bar, 100 μm. The experiment was repeated twice with similar results. TCP: tissue culture plastic. Adopted with permission from Li et al. with license number of 4595961075431 [87].

Molecular alterations of curcumin or attachment to other molecules significantly improve its activity and bioavailability, and also affect the type of differentiation. For example, the deacetylated form of curcumin has a lower inhibitory effect than the acetylated form on the adipogenic differentiation of 3T3-L1. The reason might be the hydrogen bond donor of the free phenol group in a deacetylated structure, which interferes with adipocyte differentiation [86]. According to Gupta et al., modifications of the molecular structure of curcumin are essential, as it establishes the interaction with the binding sites or receptors on the membrane of MSCs [84]. The activation or suppression of transduction signaling pathways discussed in this review, which dictate the differentiation of MSCs into each of the three mesodermal lineages, is due to the influence of the mode of curcumin interaction with the cell membrane proteins. Studies corroborated that free curcumin and curcumin associated with other biomaterials are different in terms of the effect on differentiation because incorporation into materials changes the mode of cell interactions from intracellular to interfacial. The interaction of free curcumin with binding sites on cell membranes is easier and faster than the bound curcumin, which is more persistent [87]. The physical entrapment of curcumin in silk hydrogel films and then culturing MSCs on these films resulted in an induction of adipogenic differentiation, while the same dose of curcumin when applied to MSCs in solution inhibited adipogenic differentiation and the number of lipids containing cells (Figure 5). In addition, in the case of silk-functionalized curcumin, hydrophobic molecules of curcumin interacted with hydrophobic beta-sheet domains of silk structure and induced the change in the silk secondary structure from random coil to beta sheet [87].

In a previous study, a marked improvement was shown in in vitro adipogenesis inhibition and in vivo gastrointestinal stability and bioavailability using Curcumin-34-Dichloro Phenyl Pyrazole (CDPP) compared to curcumin [88].

In a study, the inhibition of osteoclast differentiation was evaluated using gold nanoparticles functionalized with cyclodextrin curcumin complexes. In an ovariectomy (OVX)-induced osteoporosis model, the in vivo results showed significant bone density improvement and bone loss prevention by CUR-β-cyclodextrin (CD)-conjugated gold nanoparticles (CUR-CGNPs). Therefore, the CUR–CGNPs could be used as therapeutic agents for the prevention and treatment of osteoporosis [74].

In an experimental periodontitis study, effects of the oral administration of natural curcumin and a chemically modified curcumin (CMC2.24) on osteoclast-mediated bone resorption, apoptosis, and inflammation were compared in a murine model. In this in vivo study, only the number of osteoclasts and bone resorption were reduced by CMC2.24. The number of apoptotic cells in the gingival tissues and osteocytes in the alveolar bone crest were only reduced with curcumin, and not with CMC2.24 administration. CMC2.24 reduced alveolar bone resorption in the lipopolysaccharide (LPS)-induced model of periodontitis [89].

## 8. Summary and Clinical Applications

In this review article, curcumin’s effect on the mesodermal lineage differentiation of MSCs through different mechanisms and molecular pathways are discussed. Curcumin inhibits MSCs from adipogenic differentiation through modulating AMPK and activating the Wnt signaling pathway. It also inhibits Fas expression and MSCs’ proliferation, and decreases the expression of adipogenic markers through interaction with PPARγ receptors. Curcumin also interferes with the osteogenic differentiation of cells by inducing the MSCs to differentiate into osteoblasts via activating different pathways such as Akt/GSK3β, Wnt/β-catenin, Keap1/Nrf2/HO-1, and ER stress. Despite this, curcumin inhibits both osteoblast and osteoclast differentiation by suppressing the BMP/Smad and JNK/Bax signaling pathways. It also inhibits osteoclast differentiation via suppressing NF- κB and RANKL/RANK, and at the same time activates Wnt/β-catenin pathways. Furthermore, the anti-inflammatory effect of curcumin drives the chondrogenic differentiation of MSCs, while its impact on stimulating apoptosis or suppressing actin reorganization leads to the suppression of chondrogenic differentiation. The above-mentioned activities of curcumin are highly influenced by the state of curcumin (free or bound). In conclusion, it may be stated that the dose and form of curcumin can have a direct influence on its effect on mesodermal lineage differentiation, and selecting a proper mode of delivery may help in achieving the desired activity of curcumin.

With attention to the effect of curcumin on the osteogenic differentiation of cells, researchers attempted to bring curcumin into clinical use for the treatment of osteoporosis. Hatefi et al. administered 110/mg/kg/day curcumin to patients for 6 months and observed a significant increase in the bone mineral density (BMD) of the patients [40]. In another clinical study, dual-energy X-ray absorptiometry on 20 patients treated with curcumin for 12 months showed decreased bone-specific alkaline phosphatase (ALP) and C-terminal cross-linking telopeptide of type I collagen (CTx) levels and increased osteocalcin (OCN) and bone mineral density (BMD) indexes [45]. Additionally, for the treatment of osteopenia, patients received oral supplementation containing 1000 mg of phospholipidated (phytosomal) curcumin (containing 200 mg curcumin) for 6 months and increased bone density was reported [41]. Twelve months of treatment with curcumin of 110 mg/day dose combined with alendronate has been beneficial for improving the BMD score in women with osteoporosis [45].

## Figures and Tables

**Table 1:** Biomolecules involved in the mesodermal differentiation of mesenchymal stem cells (MSCs) by curcumin.

Transcription Factors	Enzymes	Protein Kinases	Apoptosis Related Genes and Proteins	Membrane or Soluble Receptors or Coreceptors	Genes	Other Proteins and Cytokines
β-catenin ATF-4 ATF-6 C/EBP Nrf2 NF-κB PPAR-γ Runx2 Osterix NFATc1 c-Fos Smad Tcf7l2 KLF15 Rex1	COX-2 HO-1 MMP9 MMP3 CPT-1 GPAT-1 IRE-1 Skp2 Cathepsin K TRAP ALP ACC TERT teP1 MnSOD	ERK JNK p38 MAPK FAK PERK Akt GSK3β Cdk PI3K AMPK	Bax Caspase-3 Fas Cidea CHOP RANKL	LRP 5 LRP6 Fz2 Wnt10b OPG DC-STAMP OSCAR	NOX4 miR-17-5p C-myc TR	BMP-2 Cyclin D1 BiP EDEM OCN OPN COL1A1 p27 Ghrelin FABP-2 IL-1β

Abbreviations: ATF: Activating transcription factor, C/EBP: CCAAT/enhancer-binding protein, Nrf2: Nuclear factor erythroid 2-related factor 2, NF-κB: Nuclear factor-kappa B, PPAR-γ: Peroxisome proliferator-activated receptor-gamma, Runx2: Runt-related transcription factor, NFATc1: Nuclear factor of activated T cells 1, Tcf7l2: Transcription factor 7-like 2, KLF15: Kruppel-like factor 15, COX-2: Cyclooxygenase-2, HO-1: Hemeoxygenase-1, MMP: Matrix metalloproteinase, CPT-1: Carnitine palmitoyltransferase-1, GPAT-1: Glycerol-3-phosphate acyl transferase-1, IRE-1: Inositol-requiring enzyme 1, Skp2: S-phase kinase-associated protein-2, TRAP: Tartrate-resistant acid phosphatase, ALP: Alkaline phosphatase, ACC: Acetylcoenzyme A carboxylase, TERT: Telomerase reverse transcriptase, teP1: Telomerase-associated protein, MnSOD: Manganese-dependent superoxide dismutase, ERK: Extracellular receptor kinase, JNK, c-jun N-terminal kinase, p38 MAPK: P38 mitogen-activated protein kinase, FAK: Focal adhesion kinase, PERK: Protein kinase R (PKR)-like endoplasmic reticulum kinase, Akt: protein kinase B, GSK3β: Glycogen synthase kinase-3β, Cdk: Cyclin-dependent kinase, PI3K: Phosphoinositide 3-kinases, AMPK: Adenosine monophosphate-activated protein kinase, Cidea: cell death inducing DFFA-like effector A, CHOP: CCAAT/enhancer binding protein (CEBP) homologous protein, RANKL: Nuclear factor κB ligand, LRP: low-density lipoprotein receptor-related protein, Fz2: frizzled, OPG: Osteoprotegerin, DC-STAMP: Dendritic cell-specific transmembrane protein, OSCAR: Osteoclast-associated immunoglobulin-like receptor, TR: Telomerase RNA subunits, BMP-2: Bone morphogenetic protein 2, BiP: Immunoglobulin-binding protein, EDEM: endoplasmic reticulum (ER) degradation-enhancing-α-mannidose-like protein, OCN: Osteocalcin, OPN: Osteopontin, COL1A1: Collagen 1, FABP-2: Adipocyte fatty acid-binding protein-2, IL-1β: Interleukin-1β.
